# Utility of the Clinical and Radiological Features in the Management of Bethesda 3 and 4 Thyroid Nodules

**DOI:** 10.1007/s13193-024-02167-7

**Published:** 2024-12-16

**Authors:** Shivakumar Thiagarajan, Swapnil Rane, Khusbhu Chandak, B. Gurukeerthi, Teja Kantamani, Vidisha Tuljapurkar, Suman Kumar, Gouri Pantvaidya

**Affiliations:** 1 Division of Head & Neck, Department of Surgical Oncology, Tata Memorial Centre and Homi Bhabha National Institute (HBNI), Mumbai, India; 2 Dept. of Pathology, Tata Memorial Centre and Homi Bhabha National Institute (HBNI), Mumbai, India; 3 Division of Head & Neck, Dept. of Surgical Oncology, Cancer Institute, Adayar, Chennai India; 4 MIOT Hospitals, Chennai, India; 5 Department of Radiology, Tata Memorial Centre and Homi Bhabha National Institute (HBNI), Mumbai, India

**Keywords:** Bethesda 3, Bethesda 4, FNAC, CUT score, Malignancy

## Abstract

Deciding the right treatment strategy for patients with Bethesda 3 and 4 fine needle aspiration cytology (FNAC) reports may be challenging. The reported risk of malignancy (ROM) and those from high-volume institutes may not be identical. The cost of molecular testing and its unavailability for routine use limits its utility in decision-making. In this study, we included all patients diagnosed at our institute with Bethesda 3 and 4 thyroid nodules on FNAC between January 2012 and December 2021. We identified the risk of malignancy for these patients along with other factors that could help identify the possibility of malignancy in these thyroid nodules. We utilised the clinical (C), ultrasound features (U), and the Bethesda category (T) to derive the CUT score and derive a cut-off value beyond which malignancy could be predicted. A total of 359 patients were included in the study. The ROM for the Bethesda 3 thyroid nodule was 77.7% (167/215), and that for the Bethesda 4 thyroid nodule was 76.4% (110/144). On multivariate analysis, nodules taller than wider [0.006, 7.662 (1.806–32.5)], male gender [0.024, 2.359 (1.119–4.976)], and the presence of microcalcification [0.004, 2.328 (1.319–4.109)] were found to be significant for the presence of malignancy in the final histopathology. The CUT score > 8.875 was associated with malignancy in the final histopathology. Various clinical and radiological factors may be useful to identify the nodules harbouring malignancy and facilitate appropriate management. The rate of malignancy in Bethesda 3 and 4 nodules among those who underwent surgery in our cohort was higher.

**Supplementary Information:**

The online version contains supplementary material available at 10.1007/s13193-024-02167-7.

## Introduction

The Bethesda System for Reporting Thyroid Cytopathology (TBSRTC) was introduced in 2007 to standardise the terminology for reporting thyroid (fine needle aspiration) cytology [1]. The Bethesda system uses six categories for thyroid cytology reporting, and a list of criteria supplements each category [1]. Each category has its risk of malignancy (ROM) and recommended management. There are variations in the risk of malignancy (ROM) across categories in the TBSRTC, first published in 2009 and subsequently revised in 2017 and 2023 [2, 3]. Also, the ROM reported in the initial publication in 2009 and then in 2017 and 2023 is variable [1–3]. The recommended management for Bethesda 3 thyroid nodule is repeated fine needle aspiration (FNA)/molecular testing/diagnostic lobectomy/surveillance, and that for Bethesda 4 thyroid nodule is molecular testing/diagnostic lobectomy [3]. There is a lot of evidence that is available favouring the use of molecular testing in these cases [4]; however, its cost and availability make them less favourable for routine use in LMICs. The ROM for Bethesda 3 and 4 thyroid nodules is important for the further management of these thyroid nodules. Also, it is important to know the ROM for all TBSRTC categories, especially for the Bethesda 3 and 4 nodules, as it has been reported to vary across institutes [5] [6] [7]. The reported ROM from high-volume institutes may not be identical to the published literature. Hence, deciding the right treatment strategy for patients with Bethesda 3 and 4 FNA reports may be challenging. Other factors (clinical and radiological) are also under consideration in the decision-making (surgery vs. observation) for Bethesda 3 or 4 thyroid nodules. Ianni and colleagues developed a scoring system (CUT score) utilising the clinical ultrasound features and the five-tiered cytological results of the FNA (T). This was done to have a cancer risk score preoperatively to reduce the number of unnecessary thyroid surgeries [8]. Heller mentioned in their article that despite all the attempts at standardisation of the nomenclature, there remains a matter of subjectivity and also that the patient population may differ from institution to institution [9]. A recommendation that each institute should attempt to identify the ROM among their patients to make an appropriate clinical decision was put forth [9]. Hence, in this study, we aimed to assess our institute’s ROM for the Bethesda 3 and 4 nodules and identify (clinical and radiological) factors that would help us predict better which Bethesda 3 and 4 thyroid nodules would harbour malignancy. We also utilised the clinical (C), ultrasound features (U), and the Bethesda category (T) to derive the CUT score [8] and derive a cut-off value beyond which malignancy could be predicted.

## Methodology

We obtained approval and waiver of the consent of the patients from our institute’s ethics committee for this retrospective study (project no. 901056). The study’s primary objective was to identify clinical and radiological factors that would help us predict which Bethesda 3 and 4 thyroid nodules would harbour malignancy. The secondary objectives were to assess the risk of malignancy in Bethesda 3 and 4 thyroid nodules at our institute and the cut-off value of the CUT score that would help predict malignancy in the Bethesda 3 and 4 thyroid nodules [8]. We also looked into the various clinical and radiological factors among patients who underwent surgery compared to those who did not undergo surgery initially in our cohort.

We included patients between January 2012 and December 2021 with Bethesda 3 and 4 nodules reported by pathologists at our institute only (the highest grade reported on FNA was taken) and those who underwent surgery subsequently, with the final histopathology report being available. We excluded patients with Bethesda 3 and 4 nodules with distant metastasis at presentation, positive (metastatic) lateral neck nodes, the index primary being in other sites with the thyroid nodule being an incidental finding, and those who were kept on observation after the diagnosis and did not undergo surgery.

The relevant demographic and clinical details such as age, gender, comorbidities, ECOG status, history of prior treatment (including head and neck irradiation), family history, clinical T-stage, N-stage, and treatment-related details such as details of the surgery and risk stratification were collected from hospital-based electronic medical records (EMR). The ultrasound details such as nodule shape (wider than taller or taller than wider), presence or absence of halo, microcalcification (presence/absence), margin irregularity, echogenicity (hypoechoic/isoechoic/hyperechoic) solid nodule or otherwise, intranodular vascularisation, nodule size (≤ 4 cms or > 4 cms), presence or absence of extrathyroidal extension, and single or multiple nodules more also recorded. The final histopathology details, including benign/malignant, type of thyroid cancer, size, focality/multicentricity, nodal metastasis, and ATA risk stratification, were also collected. All the details were collected from the electronic medical records in addition to the departmental database. The CUT score [8] was applied to these patients, and their total score was calculated. The CUT score was calculated using the clinical and ultrasound and the Bethesda category (T) in patients in whom all the findings needed to get the total score were available; it could range from 0 to 15 [8].

### Statistical Analysis

Statistical analysis was done using SPSS version 20 (IBM Corp, Armonk, New York). The univariate analysis was done to test the association for variables based on clinical relevance using the chi-square test or Fisher exact test. The multivariate analysis was done using binomial logistic regression (forward stepwise selection), and the odds ratio shall be calculated. A *p*-value of < 0.05 would be considered significant. To identify the cut-off value of the CUT score using the receiver operating curve (ROC) and Youden’s index. A nomogram was generated using the R-software, and the internal validation is to be done in an independent prospective cohort.

## Results

**Fig. 1:**
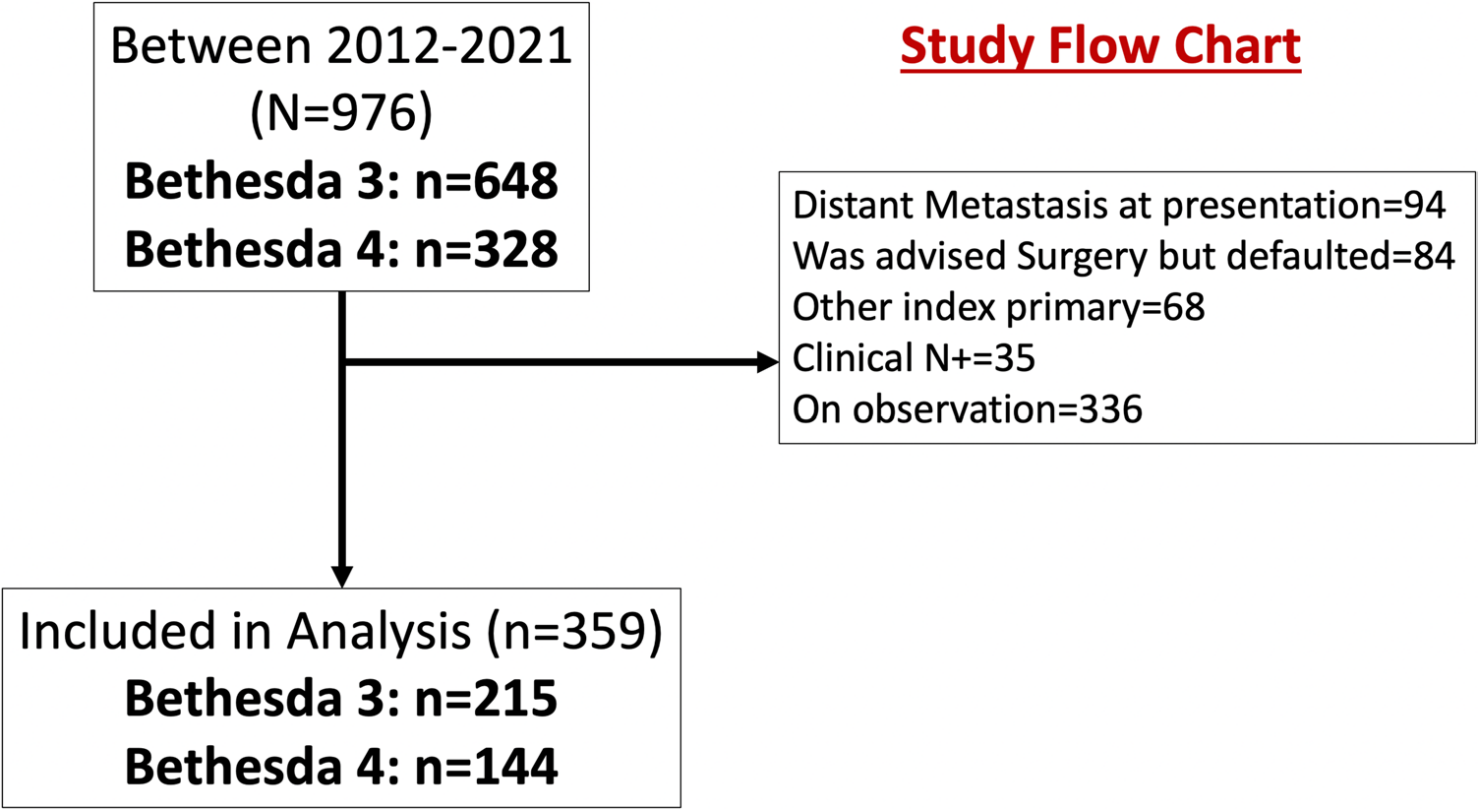
Study flow chart with details of inclusion and exclusion of the patients

A total of 976 Bethesda 3 and 4 nodules were reported between January 2012 and December 2021. Figure 1 shows the study flow chart, out of 976 Bethesda 3 and 4 thyroid nodules, 359 patients satisfied the eligibility criteria (Fig. 1) and were included in the final analysis. The ROM for the Bethesda 3 thyroid nodule was 77.7% (167/215), and that for the Bethesda 4 thyroid nodule was 76.4% (110/144). The majority of the patients were women (*n* = 294, 81.8%). Most patients underwent total thyroidectomy (*n* = 204, 56.3%), and the remaining underwent hemithyroidectomy (*n* = 155, 43.2%). Forty-eight (13.4%) patients had a pN + status following surgery. Papillary thyroid carcinoma (*n* = 224, 62.4%) was the most common type of thyroid malignancy encountered in these patients (Table 1). As per the 2015 American Thyroid Association (ATA) risk stratification [10], most patients belonged to intermediate risk (143, 39.8%) and low risk (128 (35.7%)). The various clinical and ultrasonography details, as described by Ianni and colleagues (CUT score) [8], are given in Table 2.

**Table 1:** Clinical and histopathology details

Surgery	
Hemithyroidectomy	155 (43.2%)
Total thyroidectomy	204 (56.3%)
Neck status	
pN0	228 (63.5%)
pN +	48 (13.4%)
ATA risk stratification	
Low	128 (35.7%)
Intermediate	143 (39.8%)
High	5 (1.4%)
Benign	
Follicular adenoma	14 (3.9%)
Adenoma	11 (3.1%)
Hurthle cell adenoma	18 (5%)
Lymphocytic thyroiditis	10 (2.8%)
MNG	18 (5%)
WDUMP	9 (2.5%)
Malignancy	
PTC	224 (62.4%)
FTC	24 (6.7%)
HTC	13 (3.6%)
MTC	7 (1.9%)
PDTC	8 (2.2%)
ATC	1 (0.3%)
Lymphoma	1 (0.3%)
Carcinosarcoma	1 (0.3%)

*pN0*, pathologically node-negative; *pN* + , pathologically node-positive; *MNG*, multinodular goitre; *WDUMP*, well-differentiated neoplasm of unknown malignant potential; *PTC*, papillary thyroid carcinoma; *FTC*, follicular thyroid carcinoma; *HTC*, Hurthle cell thyroid carcinoma; *MTC*, medullary thyroid carcinoma; *PDTC*, poorly differentiated thyroid carcinoma; *ATC*, anaplastic thyroid carcinoma

**Table 2:** Clinical and radiological details along with the findings of the univariate (using Chi-square test) and multivariate analysis (binomial logistic regression) to analyse the factors that predict malignancy in Bethesda 3 and 4 thyroid nodules

Clinical/USG features	Benign	Malignant	Univariate analysis *p*-value	Multivariate analysis (OR, *p*-value)
Microcalcification			0.002	0.004, **2.328**
Absent ^ref^	48	110		(1.319–4.109)
Present	28	148		
NA (23)				
Shape			0.002	0.006, **7.662**
W > T ^ref^	72	208		(1.806–32.5)
T > W	4	52		
NA (25)				
Gender			0.011	
Male	11	75		0.024, **2.359**
Female ^ref^	71	202		(1.119–4.976)
Halo sign			0.044	
Absent	24	110		
Present ^ref^	48	125		
NA (52)				
Family history			0.739	-
Yes	1	3		
No ^ref^	81	271		
NA (2)				
Prior RT			-	-
Yes	0	0		
No ^ref^	82	271		
NA (6)				
Age			0.289	-
< / = 55 years ^ref^	59	215		
> 55 years	23	62		
TSH			0.911	-
Euthyroid ^ref^	59	213		
Hypothyroid	6	20		
Hyperthyroid	4	18		
NA (39)				
Nodule margins Smooth ^ref^	68	210		
Irregular/ill-defined	9	55	0.073	-
NA (17)				
Echogenicity			0.361	-
Hypoechoic	46	168		
Others ^ref^	34	98		
NA (13)				
Nodule type			0.127	-
Solid	70	248		
Others ^ref^	10	19		
NA (12)				
Intranodular vascularisation			0.633	-
Absent ^ref^	9	25		
Present	68	230		
NA (30)				
Nodule size			0.360	-
< / = 4cms ^ref^	67	219		
> 4 cms	11	50		
NA (12)				
Nodule numbers			0.05	-
Single	58	162		
Multiple ^ref^	22	106		
NA (11)				
Vascularity			0.494	-
Central	4	17		
Peripheral ^ref^	17	46		
Both	55	193		
NA (27)				
ETE			0.05	-
Absent ^ref^	74	229		
Present	3	29		
NA (24)				

*NA*, not available (these were coded as missing data for analysis); ^*ref*^, reference variable for the analysis. *W* > *T*, wider than taller; *T* > *W*, taller than wider; *RT*, radiotherapy; *ETE*, extrathyroidal extension

**Fig. 2:**
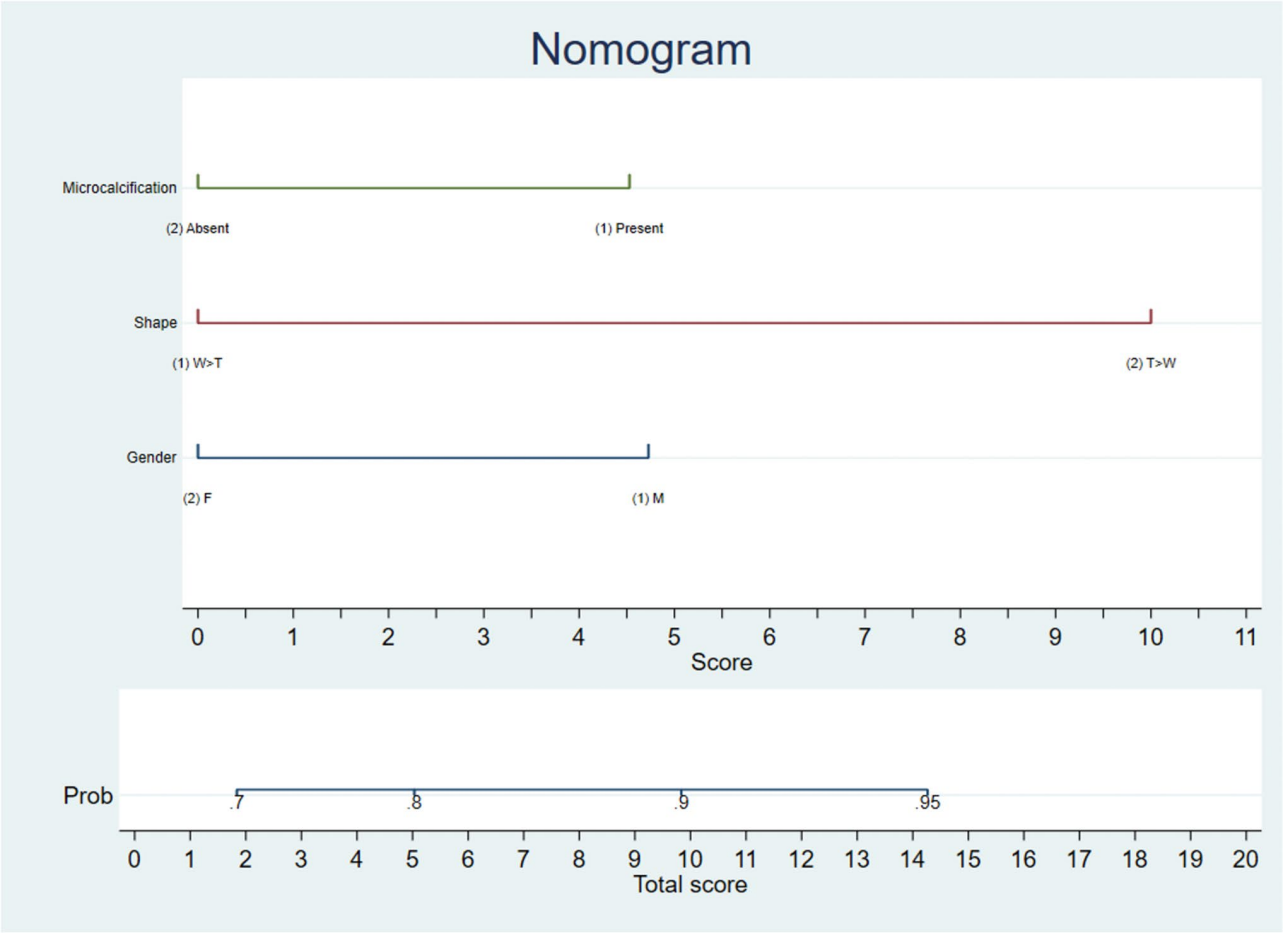
The nomogram generated based on the findings from our analysis

We performed statistical analysis to identify factors that could predict malignancy in Bethesda 3 and 4 thyroid nodules in the final histopathology (gold standard). On univariate analysis, the presence of male gender (*p* = 0.011), microcalcifications (*p* = 0.002), taller than wider (*p* = 0.002), absence of halo sign (*p* = 0.044), presence of multiple nodules (*p* = 0.05), and presence of extrathyroidal extension (*p* = 0.05) were all found to be significant (Table 2) for the presence of malignancy in the final histopathology. However, on multivariate analysis, nodules taller than wider [0.006, 7.662 (1.806–32.5)], male gender [0.024, 2.359 (1.119–4.976)], and the presence of microcalcification [0.004, 2.328 (1.319–4.109)] were found to be significant for the presence of malignancy in the final histopathology. We developed a nomogram based on the available multivariate results, and it is proposed based on a training set only (Fig. 2) (internal validation of this nomogram was done).

### CUT Score

**Fig. 3:**
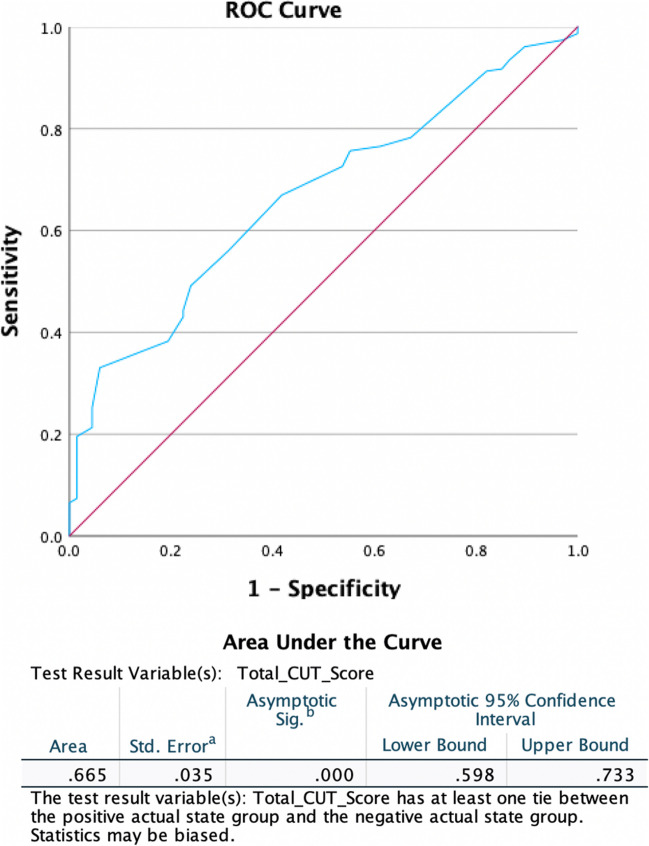
ROC for deriving the cut-off for CUT score that would help predict malignancy in the Bethesda 3 and 4 thyroid nodules

The CUT score could be obtained for 324 patients, and the median score was 7.75 (3.75–12.25). We derived a cut-off score beyond which the Bethesda 3 and 4 nodules were associated with malignancy in the final histopathology. A CUT score of > 8.875 and above was associated with malignancy in the final histopathology, with a sensitivity of 33%, specificity of 94%, and a Youden’s score of 0.27 (Fig. 3). The area under the curve (AUC) is closer to 0.7 (0.665, range 0.598–0.733) (*p* < 0.001) which can be interpreted as being acceptable.

### Surgery vs. Observation (Supplementary Table 3S)

We also tried to analyse the various clinical and radiological factors among patients who underwent surgery (*n* = 359) compared to those who did not undergo surgery initially (*n* = 336) in our cohort. We found that in our cohort, patients underwent surgery in the presence of Bethesda 4 nodules more than when it was Bethesda 3 nodules, patients older than 55 years in the presence of microcalcification on ultrasonography (USG), hypoechogenicity, > 4 cms nodules, and nodules with irregular margins.

## Discussion

The risk of malignancy (ROM) in Bethesda 3 and 4 thyroid nodules in our institute was 77.7% and 76.4%, respectively. We found that factors such as nodules that were taller than wider on ultrasonography, the presence of microcalcification, and male patients carried a higher risk of harbouring malignancy in the final histopathology. A CUT score of > 8.875 was highly suggestive of malignancy in these nodules.

The ROM in our Bethesda 3 and 4 nodules was higher than reported in the literature [3]. Ho et al. and Turkyilmaz also reported a higher percentage of malignancy in Bethesda III category nodules than expected [11, 12]. There is a > 50% prevalence of malignancy in Indian patients with indeterminate thyroid nodules who had undergone surgery [13], which is again seen in our study too. Ngo et al. reported a higher ROM among the Asian population compared to the Western population in patients with indeterminate thyroid nodules [14].

The various options for the management of Bethesda 3 and 4 thyroid nodules are ultrasound follow-ups, repeat FNA, molecular tests, and surgery [15]. Molecular testing is a well-accepted investigation for patients with Bethesda 3 and 4 thyroid nodules [4]. These molecular tests are not available uniformly across the globe [13]. The costs associated with these investigations are also unaffordable for many patients [13, 16]. Also, there is only a mild decrease in diagnostic lobectomy rates after performing these molecular tests [13]. These molecular tests are currently not done in our institute due to the costs and unavailability, as mentioned earlier. Hence, we analysed the various clinical and radiological factors that influenced our decision-making towards surgery in these patients with Bethesda 3 and 4 thyroid nodules managed at our institute.

Various studies have reported certain clinical and radiological risk factors, many of which are overlapping, associated with malignancy in Bethesda 3 and 4 thyroid nodules. Li and colleagues [17], in their systematic review of ultrasound features associated with malignancy in Bethesda 3 and 4 thyroid nodules, reported that irregular borders, solitary nodules, hypoechogenicity, microcalcifications, and taller than wider were associated with malignancy more often. Turkyilmaz et al., in their study, found that among all clinical and ultrasound features, only age > 55 years was a predictor of malignancy [12]. Liu et al. reported male gender, aspect ratio > 1, microcalcification, unclear boundary, BRAFV600E mutation, and nuclear atypia as the factors associated with malignancy in Bethesda 3 thyroid nodules [18]. Özdemir reported that the most important risk factor was hypoechogenicity of the thyroid nodule [19]. Kuru et al. reported solid structure, size ≥ 4 cm, microcalcification, hypoechogenicity, and increased vascularisation were risk factors associated with malignancy in Bethesda 3 and 4 thyroid nodules [20, 21]. Certain clinical and radiological factors can help us predict the association of malignancy in Bethesda 3 and 4 thyroid nodules, and these could be utilised in the decision-making process of the management of these thyroid nodules in the absence of availability of molecular testing.

We also utilised the CUT score reported by Ianni et al. [8] in our Bethesda 3 and 4 thyroid nodules and attempted to arrive at a cut-off score that would predict a higher risk of malignancy in our practice. Ianni et al. reported a cut-off score of > 5.25 (for all thyroid nodules) to be associated with a higher risk for malignancy (95%) with a sensitivity and specificity of 69% and 96%, respectively [8]. In our study, we derived a cut-off score for Bethesda 3 and 4 thyroid nodules in particular, and a score of > 8.875 in these Bethesda 3 and 4 thyroid nodules was highly suggestive of malignancy with a sensitivity of 33% and a specificity of 94% (this means that the ability of the cut-off score derived from identifying people without the disease is way better, i.e., the possibility of malignancy is lower if the score is less than 8.875).

The strength of our study is that it has a good sample size of exclusive Bethesda 3 and 4 thyroid nodules. It also reiterates the point that every institute must have its own risk of malignancy for the various Bethesda categories, especially Bethesda 3 and 4, which would be helpful in the decision-making. In the absence of the ready availability of molecular diagnostics, the various clinical and radiological factors and the cut-off score could be useful in the decision-making for the Bethesda 3 and 4 thyroid nodules. The limitation is the retrospective design of the study. A large number of the Bethesda 3 and 4 thyroid nodules were not operated, and their subsequent status was unknown. The nomogram needs to be validated in an external dataset.

## Conclusions

Various clinical and ultrasonography features that were significantly associated with malignancy in the Bethesda 3 and 4 nodules included nodules taller than wider, with microcalcification, and in male patients. The ROM in the operated cohort of Bethesda 3 and 4 nodules was 77.7% and 76.4%, respectively, in our study. This higher incidence of malignancy in our cohort is probably suggestive of referral bias to a tertiary cancer centre. A CUT score of greater than 8.875 was highly suggestive of malignancy in these nodules in addition to the above.

## Supplementary Information

Below is the link to the electronic supplementary material.

Supplementary file1 (DOCX 15 KB)

## Data Availability

Data may be shared if there are reasonable requests, provided local regulations are satisfied.
