# Supplementary material for: Utility of the Clinical and Radiological Features in the Management of Bethesda 3 and 4 Thyroid Nodules
Source: Indian J Surg Oncol. 2024 Dec 16;16(4):882–9. doi: 10.1007/s13193-024-02167-7 (PMC12431988; doi:10.1007/s13193-024-02167-7)
Supplement: Supplementary file 1 — Supplementary file1 (DOCX 15 KB) [file 13193_2024_2167_MOESM1_ESM.docx]

Table. 3S (Supplementary): Factors Influencing the decision of surgery (n=356) Vs observation (n=336) in the whole cohort.

| Variables | Univariate (p-Value, OR) | Multivariate (p-Value, OR) |
| --- | --- | --- |
| Bethesda category (4 vs 3) | <0.001,4.032 (2.65906.115) | <0.001, 4.080 (2.790-5.967) |
| Age (≤ 55 years vs > 55 years) | <0.001, 3.553 (2.336-5.406) | <0.001, 3.007 (2.077-4.351) |
| Microcalcification (Present vs Absent) | <0.001, 2.218 (1.514-3.248) | <0.001, 2.772 (1.898-3.905) |
| Echogenicity (Hypo vs Hyper or iso) | 0.004, 1.733 (1.197-2.510) | <0.001, 1.855 (1.296-2.654) |
| Nodule Size (≤ 4 cms vs > 4 cms) | 0.006, 2.168 (1.255-3.747) | 0.005, 2.163 (1.269-3.689) |
| Nodule margins (irregular/ill-defined vs smooth) | 0.013, 2.018 (1.161-3.505) | 0.008, 2.074 (1.207-3.561) |
| Nodule type (solid vs solid cystic/cystic) | 0.197 |  |
| Number of nodules (single vs multiple) | 0.294 |  |
| Extrathyroidal extension (present vs absent) | 0.273 |  |
| Gender (male vs female) | 0.647 |  |
| Nodule Shape (Taller than wider vs Wider than taller) | 0.138 |  |
